# The influence of three-dimensional structure on naïve T cell homeostasis and aging

**DOI:** 10.3389/fragi.2022.1045648

**Published:** 2022-11-07

**Authors:** Simon Lambert, Wenqiang Cao, Huimin Zhang, Alex Colville, Jie-Yu Liu, Cornelia M. Weyand, Jorg J. Goronzy, Claire E. Gustafson

**Affiliations:** ^1^ Department of Medicine, Veterans Administration Healthcare System, Palo Alto, CA, United States; ^2^ Division of Immunology and Rheumatology, Department of Medicine, Stanford University School of Medicine, Stanford, CA, United States; ^3^ Health Sciences Institute, Key Laboratory of Major Chronic Diseases of Nervous System of Liaoning Province, China Medical University, Shenyang, China; ^4^ Paul F. Glenn Center for Biology of Aging and Department of Neurology and Neurological Science, Stanford University School of Medicine, Stanford, CA, United States; ^5^ Allen Institute for Immunology, Seattle, WA, United States

**Keywords:** naïve T cells, immune age, homeostasis, organoids, secondary lymphoid tissues, alternative splicing

## Abstract

A breakdown in cellular homeostasis is thought to drive naïve T cell aging, however the link between naïve T cell homeostasis and aging in humans is poorly understood. To better address this, we developed a lymphoid organoid system that maintains resting naïve T cells for more than 2 weeks, in conjunction with high CD45RA expression. Deep phenotypic characterization of naïve T cells across age identified reduced CD45RA density as a hallmark of aging. A conversion from CD45RA^high^ naive cells to a CD45RA^low^ phenotype was reproduced within our organoid system by structural breakdown, but not by stromal cell aging or reduced lymphocyte density, and mediated by alternative CD45 splicing. Together, these data suggest that external influences within the lymph node microenvironment may cause phenotypic conversion of naïve T cells in older adults.

## 1 Introduction

Aging is commonly characterized by loss of effective immune responses that contributes to a higher incidence of and mortality from infection in older individuals. One key feature of immune aging is the consistent and highly reproducible decline in functional naive T cells. Naïve T cells are essential adaptive immune cells in the long-term protection against novel infections, such as SARs-CoV2, and in effective responses to vaccination (Gustafson et al., 2020b). Previous studies in older adults have shown that naive T cells not only have quantitative losses within peripheral blood and secondary lymphoid tissues (SLTs) but they also fail to maintain a truly quiescent state, demonstrating a qualitative loss in stem cell-like properties and the acquisition of a partially differentiated molecular signature (Goronzy and Cornelia, 2019). Collectively, these studies indicate that aging causes a breakdown of cellular homeostasis in naïve T cells.

Classically, naive T cell homeostasis is maintained in SLTs (e.g., tonsils, lymph nodes) by specialized stromal cells, fibroblastic reticular cells (FRCs), and their secretion of interleukin-7 (IL-7) (Link et al., 2007; Fletcher et al., 2015). However, multiple studies have found little relationship between IL-7 levels and T cell dysfunction with aging (Link et al., 2007; Marttila et al., 2011; Okoye et al., 2015). In mice, age-related changes in structural organization and cell function have been found within SLTs that may contribute to T cell dysfunction (Turner and Mabbott 2017), however the underlying cause of naïve T cell qualitative dysfunction in humans with age is unclear. Although mouse and human SLTs exhibit similar structural and cellular composition (Cakala-Jakimowicz et al. 2021), studying the longitudinal dynamics of human naïve T cell aging within tissue is difficult. In humans, access to tissue is limited and resting human naive T cells rapidly die in standard culture conditions and in most humanized mouse models. Newly-developed humanized models have been successful in reconstitution of SLTs with human T cells (Li et al., 2018). However, in the specific context of human aging and longitudinal T cell homeostasis, the mouse is not an ideal model system, as T cells in the mouse are generated mostly from ongoing thymic production in contrast to humans where this production in significantly decreased over time (Mestas and Hughes 2004; Braber et al., 2012). The development of new model systems capable of maintaining resting human naïve T cells *in vitro* is essential for improving our overall understanding of T cell homeostasis and its breakdown during aging.

Human naïve T cells are classically defined by the surface expression of CD45RA, in tandem with lymph node homing markers CCR7 and CD62L. Memory T cells on the other hand, are defined by expression of CD45RO. CD45RA and CD45RO are detected by antibodies targeting specific isoforms of CD45, a surface receptor that regulated T cell receptor signaling strength (Courtney et al., 2019). The switching of CD45 isoforms is commonly driven by T cell activation and the upregulation of splicing factors hnRNPL and hnRNPLL (Oberdoerffer et al., 2008; Preussner et al., 2012). The function of the different isoforms on specific T cell types is unclear, but decreased levels of CD45 impact a T cells’ ability to respond to antigen thus isoforms switching may also affect T cell activation potential (Wu et al., 2010).

Here, we develop a novel SLT-like organoid model system that effectively maintains human naïve T cells for more than 2 weeks, allowing for the systematic study of naïve T cell homeostasis. Using this model in conjunction with high mass cytometry, we studied the dynamics of the surface proteome of naïve T cells during aging as well as within tissue compartments to gain better insight into phenotypic changes that occur within the naïve T cell compartment in older adults. Finally, we use our SLT-like organoids to investigate potential drivers of naïve T cell aging, identifying tissue structure as a main inducer of phenotypic conversion and altered CD45RA isoform usage in older adult naïve T cells.

## 2 Materials and methods

### 2.1 Sample collection

Healthy donors (young adults, 18–35 years old; older adults, 60 years or older) were recruited through Stanford University and the Stanford Blood Center. Whole blood was collected by phlebotomy. De-identified tonsil tissues (young adults, 45 years or younger; older adults, 60 years or older) were received from the National Disease Research Interchange (NDRI). NDRI maintains a Federal Wide Assurance (FWA00006180) agreement with the DHHS, Office for Human Research Protections to comply with federal regulations concerning research involving human subjects. These studies were done in accordance with the Declaration of Helsinki. All studies were approved by Stanford University Institutional Review Boards, and all participants gave written consent prior to inclusion in these studies.

### 2.2 Subset purification

Peripheral blood mononuclear cells (PBMCs) were isolated from whole blood by Ficoll density centrifugation. CD3^+^ T cells were isolated directly from whole blood using Total T cells RosettaSep kit (Stem Cell Technologies Cat #15061). Naïve CD4 T cells were isolated from PBMCs using naïve CD4 T cell EasySep kit (Stem Cell Technologies). Stromal cells were isolated from human tonsil tissues of young or older adults using a previously published protocol (Bar-Ephraim et al., 2016). Information on tonsil donors used in FRC aging study are provided in Supplementary Table S1A. Briefly, tonsil tissue was minced and 5-10 pieces were enzymatically digested. After digestions, cells were washed and plated on collagen-coated flasks (Thermo Scientific Cat # 132707) in complete RPMI media (RPMI-1640 (Gibco) + 10% FBS + 1% Pen/Strep). After 1 day, flasks were washed with PBS to remove lymphocytes and adherent cells were grown until 90% confluent. Fibroblastic reticular cell phenotype (CD45^neg^CD31^neg^podoplanin^+^) was confirmed by flow cytometry at passage 1. Stromal cells were frozen down at passage 1 for future use.

### 2.3 Organoid generation

Organoids were modified from a previously published protocol for the use with human primary T cells (Purwada et al., 2015; Purwada and Singh 2017). Briefly, T cells and stromal cells were isolated as described above. Cells were mixed and brought up in 4% gelatin in complete RPMI media (RPMI-1640 (Gibco) + 10% FBS + 1% Pen/Strep). Based on optimization experiments, an input of 400,000 T cells and 5,000 stromal cells (1:80 ratio) were used per organoid (20 microliter total volume). Separately, 3% (w/v) biocompatible silicate nanoparticles (Laponite XLG-XR, BYK Additive Inc.) in sterile water was made and 10 µl was spotted onto 48 well, uncoated plates. Equal volume of 4% gelatin plus cell mixture was directly pipetted into the nanoparticle spots to form organoid structures for a final concentration of 2% w/v gelatin and 1.5% w/v nanoparticles. This combination creates an integrin-presenting hydrogel network *via* ionic crosslinking (Purwada et al., 2015). For 2DF conditions, 4% gelatin plus cell mixture was directly pipetted into the wells in the absence of nanoparticles. After mixing, organoids were cured for 15 min at room temperature before the addition of 400 µl complete media and incubation at 37°C. Organoids were incubated for up to 21 days, with media replacement every 7 days. For flow subsequent analysis, organoids were digested with collagenase for 30 min, prior to filtering.

### 2.4 Flow cytometry

#### 2.4.1 Surface staining

PBMCs or FRCs were stained for phenotypes using standard flow staining protocols. Antibodies are provided in Supplementary Table S2. LIVE/DEAD Fixable Aqua stain (Thermo Fisher Scientific, Cat #L34965) was used to determine live cells. For CFSE staining, cells were stained using CellTrace CFSE Cell proliferation kit (Thermo Fisher Scientific Cat #C34554) prior to addition into organoids.

#### 2.4.2 Tetramer staining

Young and older adult donors were screened for expression of HLA-A2 *via* flow cytometry. HLA-A2^+^ donors were then stained with Mart-1 26-35 (ELAGIGILTV) tetramer using previous published protocol (Rius et al., 2018). CMV pp65 495-503 (NLVPMVATV) tetramer were used as staining control. Briefly, PBMC were thawed and rested overnight at 37°C. Cells were pre-incubated with 100 nM dasatinib prior to tetramers staining (Lissina et al., 2009). After tetramer staining, cells were incubated with 5 µg anti-APC to enhance tetramer signal (Tungatt et al., 2015), followed by staining for T cell surface markers.

### 2.5 Senescence induction in stromal cells

For doxorubicin-induced senescence, FRCs were treated with 250 nM of doxorubicin (Cayman Chemical) for 24 h, followed by 83.3 nM of doxorubicin for another 48 h. Cells were assayed 7 days later. *EdU staining*: Cultured cells were pulsed with 10 mM EdU for 6.5 h in CO_2_ incubator, followed by fixation in 4% PFA for 10 min and permed in PBS + 0.5% Triton X-100 for 15 min at RT. EdU staining was performed in 0.1 M Tris-HCl (ph 7.5), 1 mM CuSO4, 0.1 M ascorbic acid and 1 µM AlexaFluor488 azide (Life Technologies) for 30 min at RT. Stained cells were washed twice with PBS + 0.5%Triton X-100 and incubated with DAPI for 5 min before immunofluorescence microscopy analysis. Positive staining was quantified in ImageJ. For RT-qPCR, cells were collected at day 14 post-induction. Total RNA and cDNA were prepared using the RNeasy Mini Kit (QIAGEN) and High Capacity cDNA RT kit (Applied Biosystems), according to manufacturers’ protocol and Taqman assays performed. Taqman assays used in these studies are listed in Supplementary Table S3.

### 2.6 Mass cytometry

Mass cytometry was performed on PBMCs by the Human Immune Monitoring Center at Stanford University. Information on donors provided in Supplementary Table S1B. Our mass cytometry panel was designed to address phenotype, activation and tissue homing properties of naïve T cells. Antibody list is provided in Supplementary Table S2. A detailed immunophenotyping protocol can be found at http://iti.stanford.edu/himc/protocols.html. All samples included normalization beads and raw data was normalized using premessa (https://github.com/ParkerICI/premessa).

### 2.7 Mass cytometry data analysis

Analyses were ran in FlowJo (V10.8). Naïve T cells were determined by hand-gating as live, single CD3^+^CD19^neg^CD14^neg^CD45RA^+^CCR7^+^ cells. To remove age-related numerical biases from subset analyses, naïve T cells were then down-sampled to equal numbers across donors (2,000 naïve CD4 or CD8 T cells per donor) prior to subsequent UMAP and Phenograph (K = 40) analyses. Naïve CD4 and CD8 T cells were analyzed separately.

### 2.8 CD45 isoform quantification

Naïve CD4 and CD8 T cells were isolated from PBMCs using EasySep isolation kits (StemCell Technologies). RNA was extracted using RNeasy Plus micro Kit (QIAGEN) and cDNA generated using VILO mastermix (Thermo Fisher Scientific). Total CD45 (gene name: PTPRC) expression was quantified using a Taqman assay (Thermo Fisher Scientific). CD45 isoforms were determined using optimized RT-PCR primers developed by Fukuhara et al. (2002). In brief, cDNA input was normalized to total CD45 expression for each sample. Normalized input was used in a 30 cycles RT-PCR using the P3 and P7 primers (Fukuhara et al., 2002). Equal amounts of RT-PCR products were ran on an agarose gel for visualization and quantified by densitometry in ImageJ.

### 2.9 Quantitative RT-PCR

Naïve CD4 and CD8 T cells were isolated fresh PBMCs using EasySep isolation kits (StemCell Technologies). RNA and cDNA was generated as described above. Gene expression was quantified using Taqman assays (Thermo Fisher Scientific) on an ABI Prism 7900HT Detection System (Applied Biosystems), and normalized to RPLP0 as a control gene. Taqman assays used in these studies are listed in Supplementary Table S3.

### 2.10 Statistical analyses

Data were analyzed using GraphPad Prism (version 9.1.0). *p*-values < 0.05 were considered statistically significant. Statistical tests used are specified in the Figure Legends.

## 3 Results

### 3.1 Resting naïve T cells are maintained by fibroblastic reticular cells within secondary lymphoid tissue-like organoids

**FIGURE 1:**
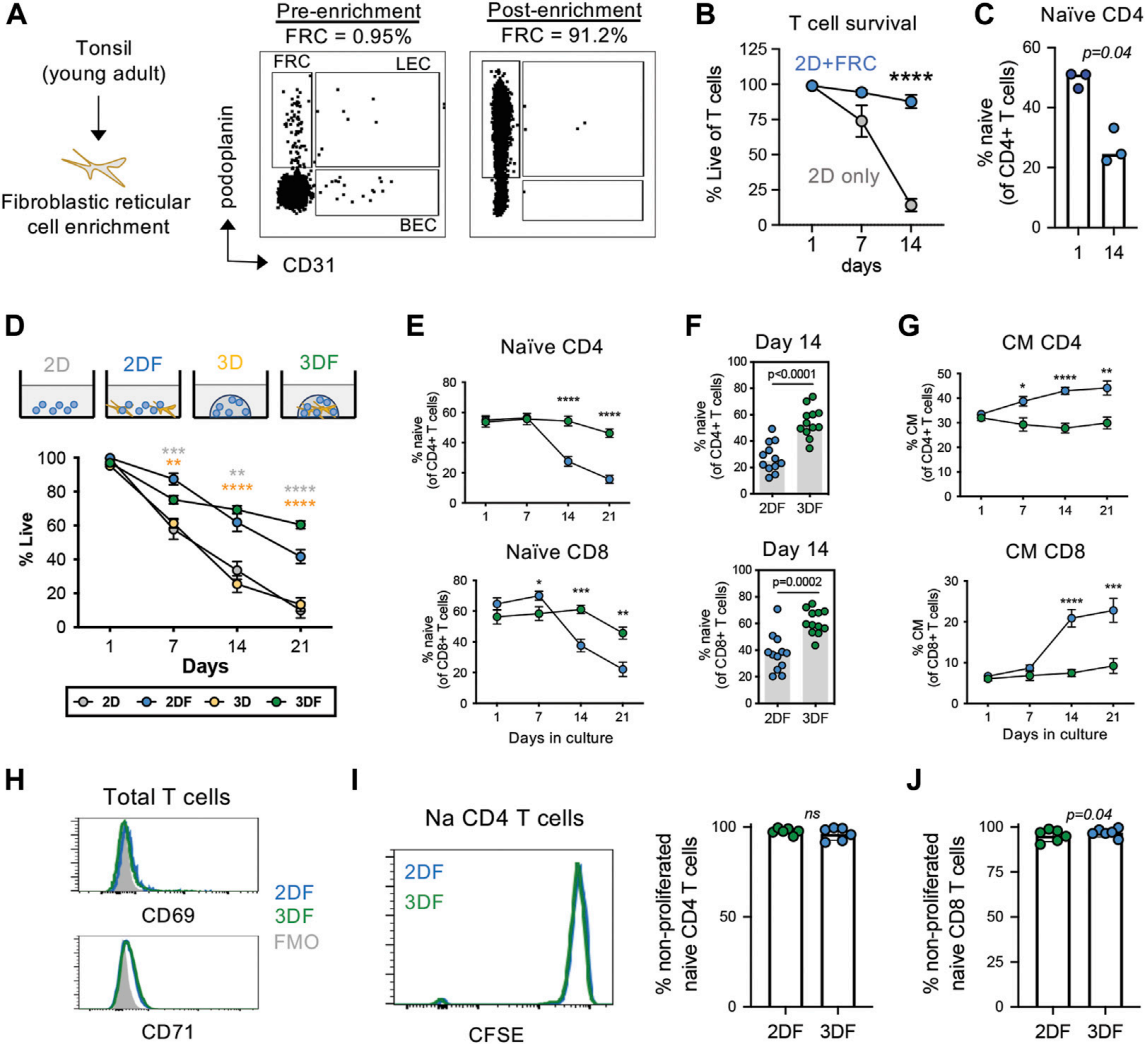
Naïve T cells are maintained in a resting state in SLT-like organoids. **(A)** Enrichment of FRCs (CD45^neg^CD31^neg^podoplanin^+^) from tonsil tissue of young adults. **(B)** Viability of total T cells and **(C)** frequency of naïve (CD45RA + CCR7+) CD4 T cells in 2D culture conditions with and without FRC co-culture (mean± SEM). (*n* = 3) **(D)** Frequencies of live T cells based on viability dye over time across all culture conditions. **(E)** Frequencies of naïve CD4 and CD8 T cells (*n* = 12) over time across 2DF and 3DF conditions. Mean ± sem. *p*-values compare 2DF vs. 3DF within one time point. **(F)** Frequencies of naïve (CD45RA + CCR7+) CD4 and CD8 T cells at day 14 in 2DF versus 3DF conditions. (*n* = 12) **(G)** Frequencies of central memory (CD45RA-CCR7+) CD4 and CD8 T cells (*n* = 12) over time across 2DF and 3DF conditions. Mean ± sem. **(H)** Expression of activation markers CD69 and CD71 on naïve CD4 T cells at day 14, representative of three individual donors. **(I,J)** CFSE proliferation analysis of **(I)** naïve CD4 T cells and **(J)** naïve CD8 T cells at day 14 (*n* = 6 young donors, paired). *p < 0.05, **p < 0.01, ***p < 0.001, ****p < 0.0001. *p*-values determined by paired *t*-test.

Human naïve T cells survive poorly under standard culture conditions in the absence of antigen stimulation, thus studying naïve T cell populations under homeostatic conditions is challenging. We initially sought to develop a model system to study naïve T cell in a “resting” condition. Because naïve T cells mainly reside within secondary lymphoid tissues, we first asked whether co-culture with T cell support cells, i.e., fibroblastic reticular cells (FRCs) isolated from SLTs, enhanced the long-term maintenance of naïve T cells *in vitro*. Primary FRCs were isolated from tonsil tissue of young adults *via* a previously published enrichment protocol (Figure 1A) and co-cultured with peripheral T cells from young donors. Although FRC co-culture significantly improved T cell survival over the course of 3 weeks (Figure 1B), the frequencies of CD45RA + CCR7+ naïve T cells rapidly declined over this same time course (Figure 1C). This suggests that although FRC provide survival factors to T cells, they are un-able to maintain naïve T cell homeostasis in standard cell culture conditions.

Three-dimensional (3D) culturing has proven useful for growth of many cell types. This led us to next investigate the potential of an organoid-based model system for studying T cell homeostasis. We focused on nanoparticle-based organoid systems for the maintenance of naïve T cells over time, as these systems are more malleable than other newer tissue-based systems in the context of aging (Wagar et al., 2021). Using this system, we first compared T cell survival in organoids (3DF) with that of 2D culturing in the presence of FRCs (2DF) (Figure 1D). Both 2DF and 3DF conditions maintained relatively high T cell viability (median 62% vs. 69% at day 14, respectively) (Figure 1E). 2D and 3D culturing of T cells alone (i.e., without FRCs) demonstrated rapid loss of cell viability over the same time course (25% and 33% at day 14, respectively), and thus are excluded from subsequent phenotypic analyses. An example of image of 3D and 3DF organoids is provided in Supplementary Figure S1. Notably, unlike the rapid decline in 2DF conditions, the frequencies of naïve T cells stayed significantly higher and were maintained longer in organoids (Figures 1E,F), with naïve CD4 T cells showing slightly better maintenance than CD8 T cells over a 3-week time course.

Homeostatic maintenance requires cell survival in the relative absence of activation, proliferation and/or differentiation. Thus, we next assessed the status of these three features in 3DF organoids compared with 2DF conditions. Firstly, when undergoing differentiation, naïve T cells transition into memory phenotypes. In association with the decrease in naïve T cells we observed in 2DF, we found a significant increase in central memory cells in 2DF compared with 3DF organoids, indicating 3DF prevents naïve T cell differentiation (Figure 1G). However, after 14 days of culturing, there was no significant expression of activation markers CD69 and CD71 on naïve CD4 T cells in either 2DF or 3DF condition, implying limited amounts of on-going T cell activation (Figure 1H). Similar negative results were found at day 1 and day 7. Additionally, little to no cell proliferation was observed within the naïve compartment in 2DF or 3DF across a 14 day time course, >95% of naïve T cells remaining in a non-proliferated state (CFSE^high^) (Figures 1I,J). Thus, SLT-like organoids can maintain naïve T cells in a resting, homeostatic state for at least 2 weeks, whereas 2-dimensional co-culturing leads to T cell pseudo-differentiation within 7 days, even in the absence of cell activation or proliferation.

### 3.2 Organoids maintain a unique CD45RA^high^ naïve cell phenotype, preventing the acquisition of common T cell aging characteristics

**FIGURE 2:**
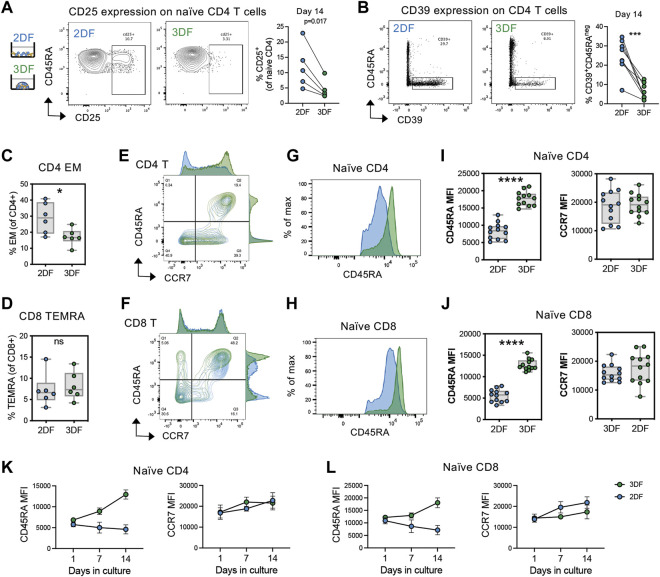
SLT-organoids prevent T cells from acquiring features of immune aging and naïve cell-specific decline in CD45RA expression. **(A)** CD25^+^ naïve CD4 T cell frequencies in naïve CD4 T cells in 2DF and 3DF (*n* = 5 young adult T cell donors) after 14 days of culturing. **(B)** CD39^+^CD45RA^neg^ CD4 T cells frequencies in total CD4 T cells in 2DF and 3DF (*n* = 8 young adult T cell donors) after 14 days of culturing. **(C)** Effector memory (CD45RA^neg^CCR7^neg^) CD4 T cell and **(D)** TEMRA (CD45RA^+^CCR7^neg^) CD8 T cell frequencies in 2DF and 3DF (*n* = 6 young adult T cell donors) after 14 days of culturing. **(E,F)** Distribution of **(E)** CD4 and **(F)** CD8 T cells by CD45RA and CCR7 expression after 14 days of culturing. **(G,H)** Representative histograms of CD45RA expression on naïve (CD45RA + CCR7+) **(G)** CD4 and **(H)** CD8 T cells in 2DF and 3DF after 14 days of culturing. **(I,J)** CD45RA and CCR7 MFI on naïve **(I)** CD4 and **(J)** CD8 T cells in 2DF and 3DF (*n* = 6 young adult T cell donors done in duplicate) after 14 days of culturing. **(K, L)** CD45RA and CCR7 MFI over time on naïve **(K)** CD4 and **(L)** CD8 T cells in 2DF and 3DF (n = 6 young adult T cell donors done in duplicate). *p < 0.05, **p < 0.01, ***p < 0.001, ****p < 0.000. *p*-values determined paired *t*-test.

During aging, naïve T cells numerically decline with a reciprocal expansion in the memory compartment. Previous works determined that CD45RA + CCR7+ naïve T cells take on transcriptional and epigenetic features of memory T cells with aging (Moskowitz et al., 2017; Hu et al., 2020). Thus, we wanted to further investigate the acquisition of age-related phenotypes of T cells in organoids compared with 2DF culturing as a model for studying T cell aging. Consistent with previous literature on *ex vivo* T cell aging (Pekalski et al., 2013), we found that naïve CD4 T cells acquired elevated levels of CD25 in 2DF, however this increase was prevented in our SLT-like organoids (Figure 2A). Moreover, we see increased CD39^+^ memory T cells, another feature of T cell aging (Fang et al., 2016)in 2DF, was again prevented in the organoids (Figure 2B). We additionally see increased effector memory CD4 T cells but not CD8 terminal differentiated effector (TEMRA) cells in 2DF compared with organoids (Figures 2C,D), consistent with CMV but not age specifically driving expansion of the TEMRA population. Further characterization of the naïve compartment also revealed a consistent downregulation of CD45RA expression on both naïve CD4 and CD8 T cells in 2DF but not in the organoids (Figures 2E–H). This decrease in CD45RA at day 14 was detected across multiple experiments (Figures 2I,J). Conversely, CCR7 expression was unchanged at day 14. CD45RA expression levels steadily decreased over time in 2DF, whereas overall CD45RA MFI increased over the same time course in the organoids (Figures 2K,L). Collectively, these data demonstrate that our SLT-organoid prevents some age-related changes of T cells observed *ex vivo* in the periphery and indicates the decreased density of CD45RA as a potential new marker of naïve T cell aging and homeostatic breakdown.

### 3.3 CD45RA^high^ naïve T cells convert to CD45RA^low^ phenotype with age

**FIGURE 3:**
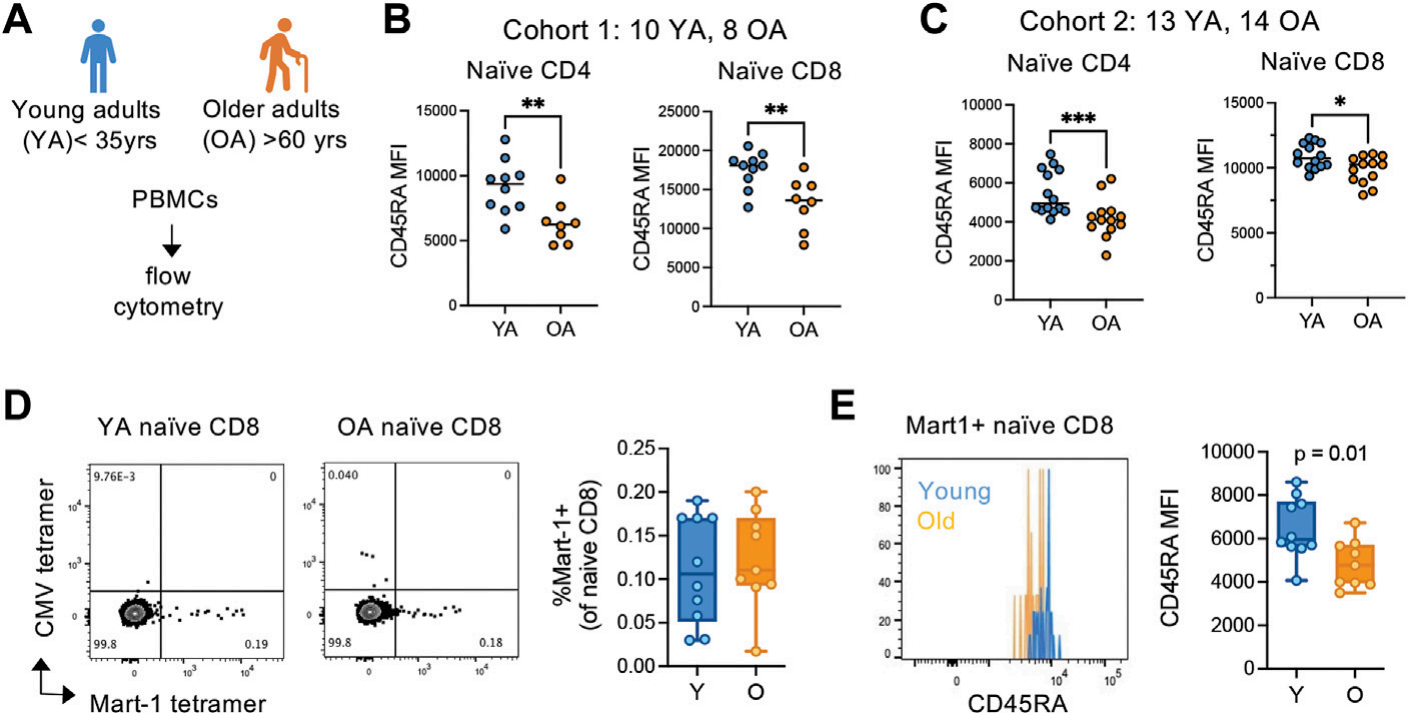
CD45RA expression is globally reduced on antigen-inexperienced naïve T cells in older adults. **(A)** Overview of young (YA; <35 years old) and older (OA; >60 years old) adult cohort design for peripheral blood mononuclear cell flow cytometry. Flow analysis was done same day, same instrument for each independent cohort. **(B)** Cohort 1 (YA = 10, OA = 8) and **(C)** cohort 2 (YA = 13, OA = 14) analysis of CD45RA expression on the surface of naïve CD4 and CD8 T cells. **(D)** Frequencies of and **(E)** CD45RA MFI on Mart-1-specific naïve CD8 T cells from young (*n* = 10) and older (*n* = 9) HLA-A2^+^ adults. CMV pp65 495-503 (NLVPMVATV) tetramer was used as a control for non-specific binding. *p < 0.05, **p < 0.01, ***p < 0.001. ns, not significant. *p*-values determined by Mann-Whitney test.

Whether downregulation of CD45RA expression is a feature of naïve T cell aging is unknown. Thus, we initially examined the relative density of CD45RA on naive (CD45RA + CCR7+) T cells in two independent cohorts of young (35 years or less) and older (60 years or greater) adult PBMCs using flow cytometry (Figure 3A). All samples from each cohort were ran on the same day and the same instrument to be able to compare relative expression levels of CD45RA across samples. We found a significant and reproducible decrease in CD45RA on both naïve CD4 and CD8 T cells from older adults compared with young adults (Figures 3B,C), suggesting that the decrease in CD45RA MFI is caused by homeostatic breakdown in the aging process.

The breakdown of cellular quiescence and adaptive acquisition of memory-like features are thought to be driving features of naïve T cell aging. Thus, we next wanted to determine whether the decrease in CD45RA in naïve T cells with age is caused by cellular adaptation, as suggested by our SLT-like organoid data. To delineate whether naive cells are converting to alternative phenotypes *in vivo* with age, we examined *ex vivo* phenotypes of antigen-inexperienced naive cell populations by Mart-1 (melanoma antigen) tetramer staining in HLA-A2+ melanoma-naïve young (*n* = 10) and older (*n* = 9) adults. Firstly, we found that young and older adults have similar frequencies of Mart-1+ naïve (CD45RA + CCR7+) CD8 T cells within the naïve compartment, albeit older adults have numerically fewer cells (Figure 3D). However, the density of CD45RA was significantly reduced on Mart-1+ naïve CD8 cells from older adults compared with young adults (Figure 3E). Because these cells are from melanoma-naïve individuals, these data indicate the naïve compartment globally converts towards a CD45RA^low^ phenotype with age, independent of antigen-experience.

### 3.4 A unique CD45RA^high^CD27^high^CD38^+^CD25^neg^ naïve T cell subset is lost in the periphery with age

**FIGURE 4:**
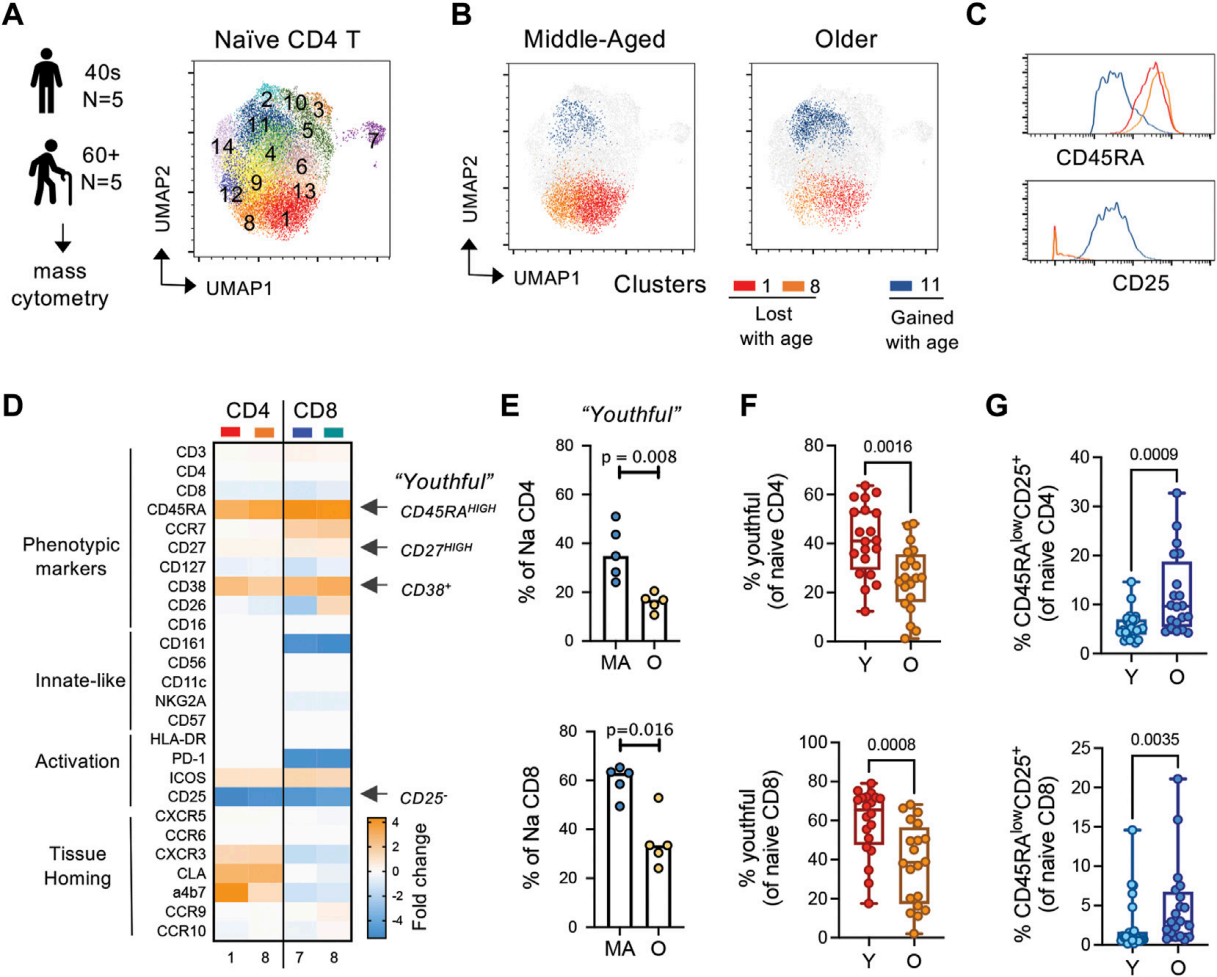
CD45RA^high^ subset of naïve T cells is replaced by a CD45RA^low^, CD25^low/+^ subset with age. **(A)** General experimental outline and Phenograph clustering of naïve CD4 T cells from middle-aged (MA; *n* = 5; 40–49 years) and older (OA; *n* = 5; >60 years) adults. **(B)** UMAP of differential clusters between MA and OA naïve CD4 T cells. **(C)** Histograms of select markers delineating age-related clusters. **(D)** Relative markers expression in naïve CD4 and CD8 clusters that decrease with age compared with those that increase with age. **(E)** Hand-gated frequencies of population lost with age (termed “youthful;” CD45RA^high^CD27^high^ CD38^+^CD25^neg^ naïve T cells). **(F,G)** Frequencies of **(F)** youthful and **(G)** CD45RA^low^CD25^+^ naïve T cells in a follow-up cohort of young (*n* = 20; <35 years) and older (*n* = 20; >60 years) individuals. *p*-values determined by Mann-Whitney test.

We next hypothesized that the CD45RA^high^ population of naïve T cells is a unique subset of naïve T cells and this subset is lost with age. Thus, we next sought to further characterize, in an unbiased fashion, phenotypically distinct subsets within CD45RA + CCR7+ naïve T cells in “healthy” middle-aged (*n* = 5, 40–50 years) and older (*n* = 5, >60 years) adults using mass cytometry (Figure 4A) Our mass cytometry panel was designed to address basic phenotype, activation and tissue homing properties of T cells (Supplementary Table S2). An overview of the mass cytometry data analysis is provided in Supplementary Figure S2A. For our analysis, we normalized our input to equal cell number for each compartment across age (2,000 cells per naïve compartment per donor). Unsupervised clustering analysis *via* Phenograph identified 14 clusters within the naïve CD4 T cells with three major clusters that changed with age; cluster 1 and 8 decreased and cluster 11 increased with age (Figure 4B; Supplementary Figure S2B).

To better understand the phenotypic differences between age-biased clusters in naïve CD4 T cells, we next compared the marker expression profiles within these clusters. In the CD4 compartment, age-decreased clusters 1 and 8 were differentiated mainly by expression of the homing marker alpha4beta7 (Supplementary Figure S2C). Notably, compared to the age-increased cluster 11, age-decreased clusters 1 and 8 displayed high expression of CD45RA and CD27, were CD38 high-positive and CD25 negative (Figures 4C,D; Supplementary Figure S2C). This high expression of CD45RA on naïve T cells in middle-aged adults is consistent with high expression of CD45RA maintained on naïve T cells in our organoids and in young adults in our initial flow cytometry cohorts (Figures 2, 3). The age-increased cluster 11 expressed lower levels of CD45RA and CD27 compared with clusters 1 and 8, had mixed CD38 expression and low-positive expression of CD25. A similar comparison of the naive CD8 compartment showed that this compartment was much more significantly affected by age, with 7 out of 15 clusters showing age-related differential expression (2 clusters decreased and 5 clusters increased with age) (Supplementary Figures S2D,E). Moreover, we found that both the CD4 and CD8 compartment shared parallel changes in phenotypic features across age, with middle-aged naïve compartment being enriched in CD45RA^high^CD27^high^CD38^+^CD25^neg^ cells (Figure 4D; Supplementary Figures S2F,G). Back-gating of our mass cytometry data similarly revealed that this CD45RA^high^CD27^high^CD38^+^CD25^neg^ subset of naïve cells was decreased with age (Figure 4E). Moreover, in a larger follow-up cohort of young (<35 years, *n* = 20) and older (>60 years, *n* = 20) adults, we observed that the CD45RA^high^CD27^high^CD38^+^CD25^neg^ population within CD45RA + CCR7+ naïve CD4 and CD8 T cells was significantly decreased with age (Figure 4F). Based on their decline with age, we termed these cells as “youthful.” Gating strategy for flow cytometry analysis is provided in Supplementary Figure S3. We also found higher frequencies of CD45RA^low^CD25^+^ naïve CD4 and CD8 T cells with age (Figure 4G), again in line with the aging literature and our age-related organoid model. Thus, using a combination of our organoid model system, high-dimensional phenotypic analysis, and classical flow cytometry techniques, we discovered novel phenotypic changes in naïve T cells with aging—particularly noted by the downregulation of CD45RA and CD27 on a subset of cells.

### 3.5 CD45RA^high^ naïve T cells are preferentially maintained in lymph nodes and secondary lymphoid tissue-like organoids

**FIGURE 5:**
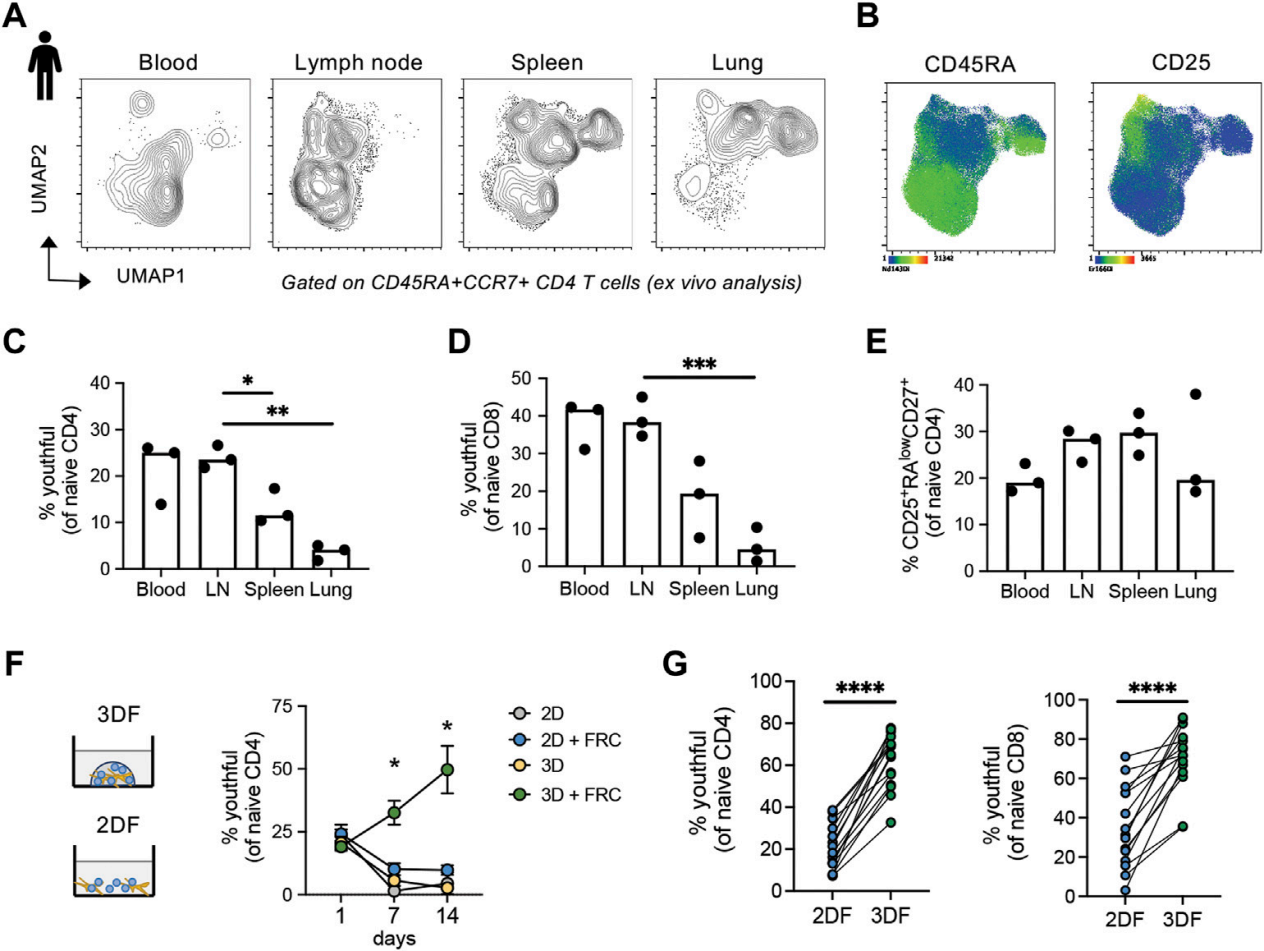
CD45RA^high^ naïve T cells are enriched in human blood, lymph nodes and SLT-like organoids. **(A)** UMAP of naïve CD4 T cells across different tissue types from young adults (*n* = 3). **(B)** Markers expression within naïve CD4 T cells from tissues. **(C–E)** Frequencies of **(C)** CD45RA^high^CD27^high^CD38^+^CD25^neg^ and **(D)** CD25^+^CD45RA^low^CD27^low^ naïve CD4 and **(E)** CD45RA^high^CD27^high^CD38^+^CD25^neg^ naïve CD8 T cells across tissue type. Mass cytometry dataset is from publicly available source ImmPort SDY1389. **(F)** Frequencies of CD45RA^high^CD27^high^CD38^+^CD25^neg^ naive CD4 T cells over time across different culture conditions (*n* = 3 young donors). **(G)** Frequency of CD45RA^high^CD27^high^ naïve CD4 and CD8 T cells in larger cohort of young donors (*n* = 15) at day 14 in either 2DF or 3DF conditions. *p < 0.05, **p < 0.01, ***p < 0.001, ****p < 0.0001. *p*-values were determined by **(C–E)** one-way ANOVA and **(F,G)** paired *t*-test.

Although naïve T cells circulate through the blood, their primary residence is within lymph node and secondary lymph node structures. Maintenance of CD45RA^high^ naïve T cells within our organoids suggests that these cells may specifically reside in tissue-like environments. Thus, we wanted to determine the tissue localization of the “youthful” CD45RA^high^ naïve T cell subsets and whether these cells preferentially reside in lymph nodes. Utilizing an available mass cytometry dataset from young adults (ImmPort SDY1389), we interrogated the phenotypic features of naïve (CD45RA^+^CCR7^+^) CD4 T cells within four different tissues; blood, lymph node, spleen and lung. Using UMAP analysis, we found that naïve CD4 T cells from blood were most similar to those in lymph node, but relatively distinct from spleen and lung (Figure 5A). The naïve CD4 T cells most prevalent in blood and LN also had higher expression of CD45RA (Figure 5B). Hand-gating on the naïve populations revealed that a large proportion of the naïve T cells in blood and LN tissues of young adults were of the youthful (CD45RA^high^CD27^high^CD38^+^CD25^neg^) phenotype (Figure 5C). Similar to the CD4 compartment, youthful naïve CD8 T cells were also found in higher frequencies in lymph nodes and blood (Figure 5D). The same enrichment was not seen for the age-related CD45RA^low/+^ CD27^+^CD25^+^ naïve CD4 populations, which displayed little tissue-bias (Figure 5E). Thus, youthful CD45RA^high^ naïve T cells appear to be a lymph node-enriched population of naïve cells and highlights the importance and potential functionality of our SLT-like organoid in the study of human naïve T cell homeostasis.

To determine whether the CD45RA^high^ naïve T cells maintained within our SLT-like organoids are also the LN-enriched youthful subset, we examined frequencies of CD45RA^high^CD27^high^CD38^+^CD25^neg^ cells within the naïve CD4 T cell compartment over time. Notably, we found expansion of the youthful subset within naïve T cells out to 14 days, compared with a rapid loss of this subset in all other conditions (Figure 5F). Repeat experiments using CD45RA^high^CD27^high^ as the definition for youthful also confirmed that this population in both CD4 and CD8 naïve T cells is expanded within SLT-like organoids at 2 weeks post-culturing compared with 2DF conditions (Figure 5G). Thus, SLT-like organoids not only maintain total CD45RA + CCR7+ naïve T cells, but they also preferentially maintain naïve T cells in a youthful state, consistent with the enrichment of this naïve population within lymph nodes.

### 3.6 A conversion of CD45RA^high^ to CD45RA^low^ phenotype is not caused by fibroblastic reticular cell aging

**FIGURE 6:**
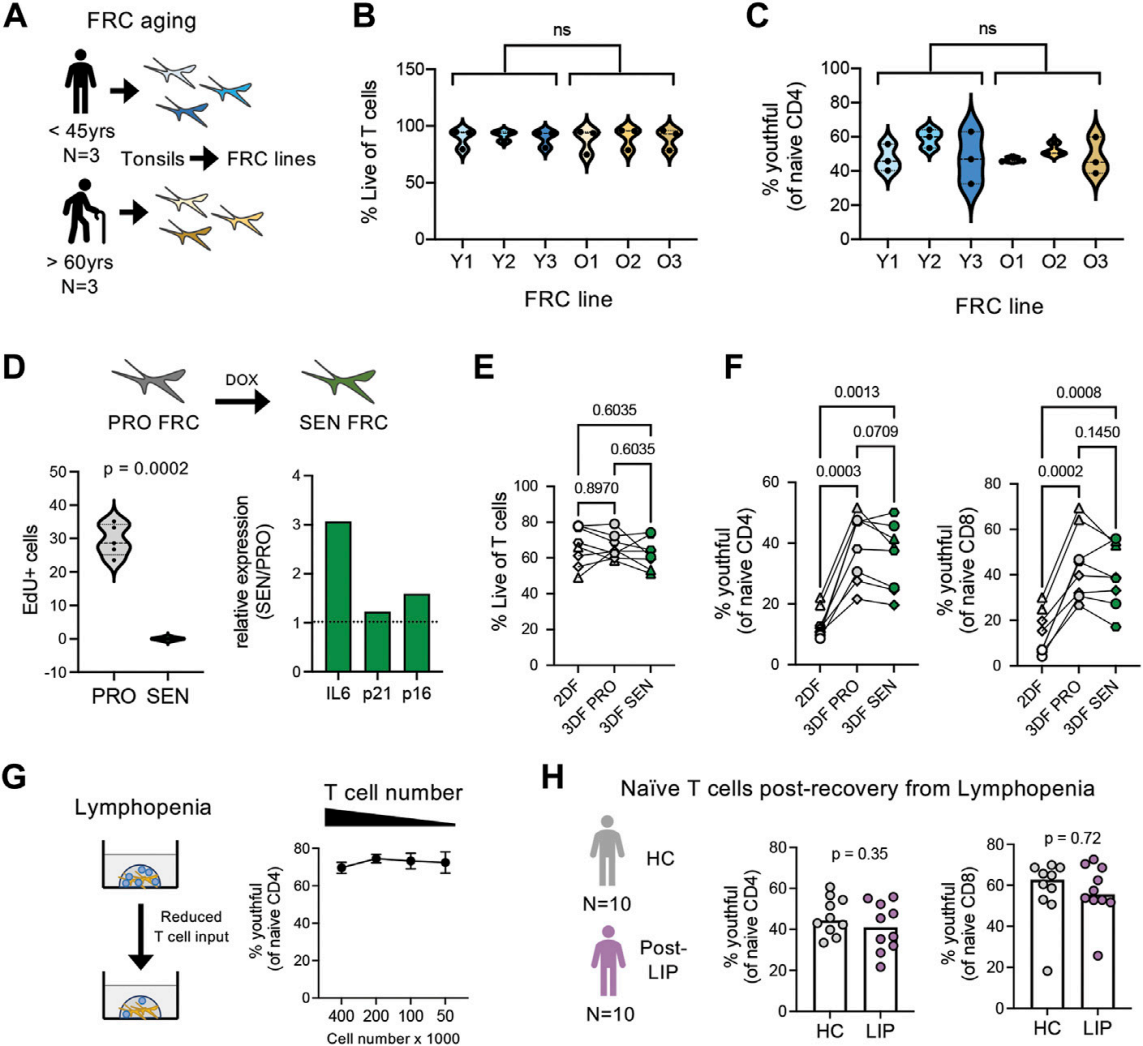
Effects of FRC aging, senescence, and lymphopenia on naïve T cell aging. **(A)** Overview of fibroblastic reticular cell isolation from young (*n* = 3; 27, 31, and 41 years old) and older (*n* = 3; 85, 94, and 96 years old) adults. **(B)** T cell viability and **(C)** CD45RA^high^CD27^high^ naïve CD4 T cells after 14 days organoid cultures (*n* = 3 young T cell donors) with FRCs isolated from young or older adults. **(D)** Overview and characterization of senescent FRCs generated from FRCs from young tissue donor. Relative gene expression of IL-6, p21 and p16 determined by qPCR, normalized to RPLP0 control gene. **(E)** Percent Live T cells and **(F)** frequencies of CD45RA^high^CD27^high^ naïve CD4 and CD8 T cells in 2DF (comparison control) or 3DF with proliferating (PRO) or senescent (SEN) FRCs. T cells are from four different young donors, performed in duplicate. **(G)** Effect of T cell input number on CD45RA^high^CD27^high^ naïve CD4 T cell frequencies in young donors (*n* = 4) after 14 days organoid cultures. **(H)** Frequencies of CD45RA^high^CD27^high^CD38^+^CD25^neg^ naïve CD4 and CD8 T cells in cohort of patients 1–2 years after chemotherapy induced lymphopenia (*n* = 10) compared with control cohort (*n* = 10). ns, not significant. *p*-values determined by **(B,C)** paired *t*-test, **(E–G)** RM one-way ANOVA with Holm-Sidak multiple comparisons tests and **(H)** Mann-Whitney test.

Using the combination of our novel model for naïve T cell homeostasis and new phenotypic definition for youthful naïve T cells, we wanted to interrogate possible mechanisms driving naive T cell aging. Because FRCs play such a pivotal role in the maintenance of naïve T cells within our organoids as well as *in vivo* within lymph nodes, we first asked whether FRC functional differences with age may affect naïve T cell aging. For these studies, we isolated FRC cell lines from tonsils of three young (<45 years) and three older (>60 years) adults and compared the ability of these cell lines to maintain the youthful state of naïve T cells (Figure 6A). However, all six FRC cell lines demonstrated similar T cell survival over 14 days (Figure 6B). Moreover, although there was some variation across FRC cell lines, no difference in the frequency of youthful naïve T cells was found between FRC lines by age (Figure 6C). Thus, FRCs appear to remain functional across age.

One unique feature of aging is the accumulation of senescent cells (Gorgoulis et al., 2019). However, our FRC isolation protocol excludes non-proliferating cells from isolation. Thus, we next wanted to address whether introduction of FRC senescence could model T cell aging. For these studies, we generated senescent (SEN) FRCs from young donor FRC lines *via* chemotherapeutic agents, which inhibit FRC cell proliferation and promote SASP (Figure 6D). Similar to the FRC aging studies, SEN FRCs exhibited a comparable capacity as proliferating (PRO) FRCs to maintain T cell survival and youthful naïve T cell frequencies, albeit a non-significant trend for decreased frequencies of youthful naïve CD4 T cells with SEN FRCs (Figures 6E,F). Together, these data suggest that FRCs ability to maintain naïve T cell homeostasis is not directly affected by age or closely modeled by the introduction of stromal cell senescence.

Immune aging is linked with lymphopenia. Thus, we next studied the potential effect of lymphopenia on naïve T cell aging and youthful conversion. Reducing the initial cell numbers within SLT-like organoids did not affect the stability of youthful naïve T cells (Figure 6G). Likewise directly *ex vivo*, we did not find a significant difference in youthful frequencies in a cohort of middle-aged breast cancer patients, who had received lymphopenia-inducing chemotherapy 1-2 years prior to blood draw (Gustafson et al., 2020a), compared with age- and sex-matched healthy controls (Figure 6H). Thus, re-population after lymphopenia itself does not appear sufficient to induce phenotypic conversion of naïve T cells.

### 3.7 3D structural breakdown but not lymphopenia cause a conversion of CD45RA^high^ to CD45RA^low^ phenotype

**FIGURE 7:**
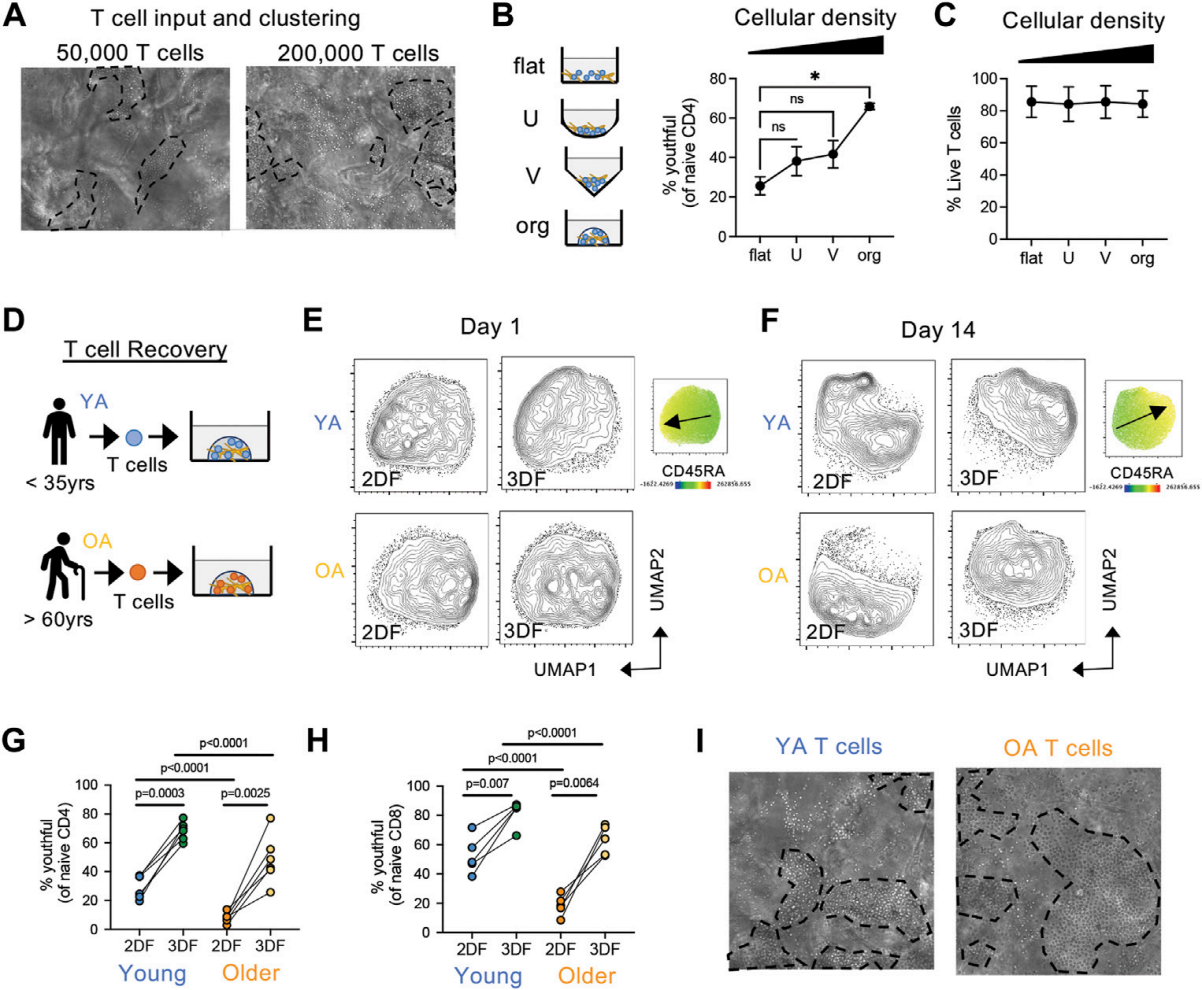
Structure and cell density effect naïve T cell phenotype and the conversion from CD45RA^high^ to CD45RA^low^. **(A)** Clustering of T cells in organoids at different cell densities after 7 days. Representative of three independent experiments. **(B,C)** Effect of cell density/contact capability on **(B)** CD45RA^high^CD27^high^ naïve CD4 T cells and **(C)** T cell viability in young donor T cells (*n* = 3). T cells were cultured with FRCs (from young adult) in flat, U-bottom, V-bottom or organoids for 14 days. **(D)** T cells were isolate from young (YA; 35 years or less) and older (OA; 60 years or older) adult peripheral blood and cultured in organoids with FRCs (from young adult). **(E,F)** Analysis of naïve CD4 T cell similarly at **(E)** day 1 and **(F)** day 14 post-culturing. CD45RA expression map is on the right with arrow indicating low to high expression. **(G,H)** Frequencies of CD45RA^high^CD27^high^ **(G)** CD4 and **(H)** CD8 naïve T cells from young (*n* = 6 CD4, *n* = 5 CD8) and older (*n* = 6 CD4, *n* = 5 CD8) donors. **(I)** Clustering of T cells from young or old donors in organoids. Representative of more than three independent experiments. *p < 0.05 *p*-values were determined by **(B,C)** Friedman test with Dunn’s multiple comparison test, and **(G,H)** Mixed-effects analysis with Holm-Sidak multiple comparison.

During these experiments, we noticed that regardless of cell input number, T cells formed tight clusters within organoids (Figure 7A), suggesting that T cell clustering is critical for youthful maintenance. To determine whether close T cell contact in the presence of FRCs is sufficient for youthful maintenance, we co-cultured T cells with FRCs in flat, U-bottom or V-bottom plates and compared youthful frequencies over time to that of organoids. We found that although youthful cells trended higher in U-bottom and V-bottom plates, organoid culturing maintained youthful naïve T cells significantly better than any of the 2D conditions (Figure 7B). Of note, all conditions had similar cell viability (Figure 7C). Thus, the ability of naïve T cell to maintain a youthful state requires T cell clustering in a structured 3D space, regardless of absolute cell number. The aging microenvironment is known to have a breakdown in structure with age (Thompson et al., 2017), therefore we next hypothesized if an aged naïve T cells from older individuals was placed back into structured environment (i.e., our SLT-like organoid) they may revert back to a youthful state. Thus, we compared the enrichment of youthful naïve T cells in young and older adults (Figure 7D). Of note, naive CD4 T cells from older adults are mainly CD45RA^low^ at day 1, regardless of culturing condition (Figure 7E). However, naïve CD4 T cells from older individuals, which mainly had a CD45RA^low^ phenotype at input, showed significantly increased frequencies of youthful CD45RA^high^ cells after 14 days in a 3D structure, albeit at a lower level than those from young donors (Figures 7F,G). Youthful naïve CD8 T cells showed the same increase (Figure 7H). Moreover, these results are consistent with the robust cell clustering we observed in T cells from both age groups in our SLT-organoids (Figure 7I). Together, these data imply that naïve T cells require a structured microenvironment for homeostatic maintenance and that a breakdown in LN structure can cause the conversion of naïve T cells from a CD45RA^high^ to a CD45RA^low^ phenotype.

### 3.8 Age-related conversion of naïve cells from CD45RA^high^ to CD45RA^low^ is caused by alternative splicing

**FIGURE 8:**
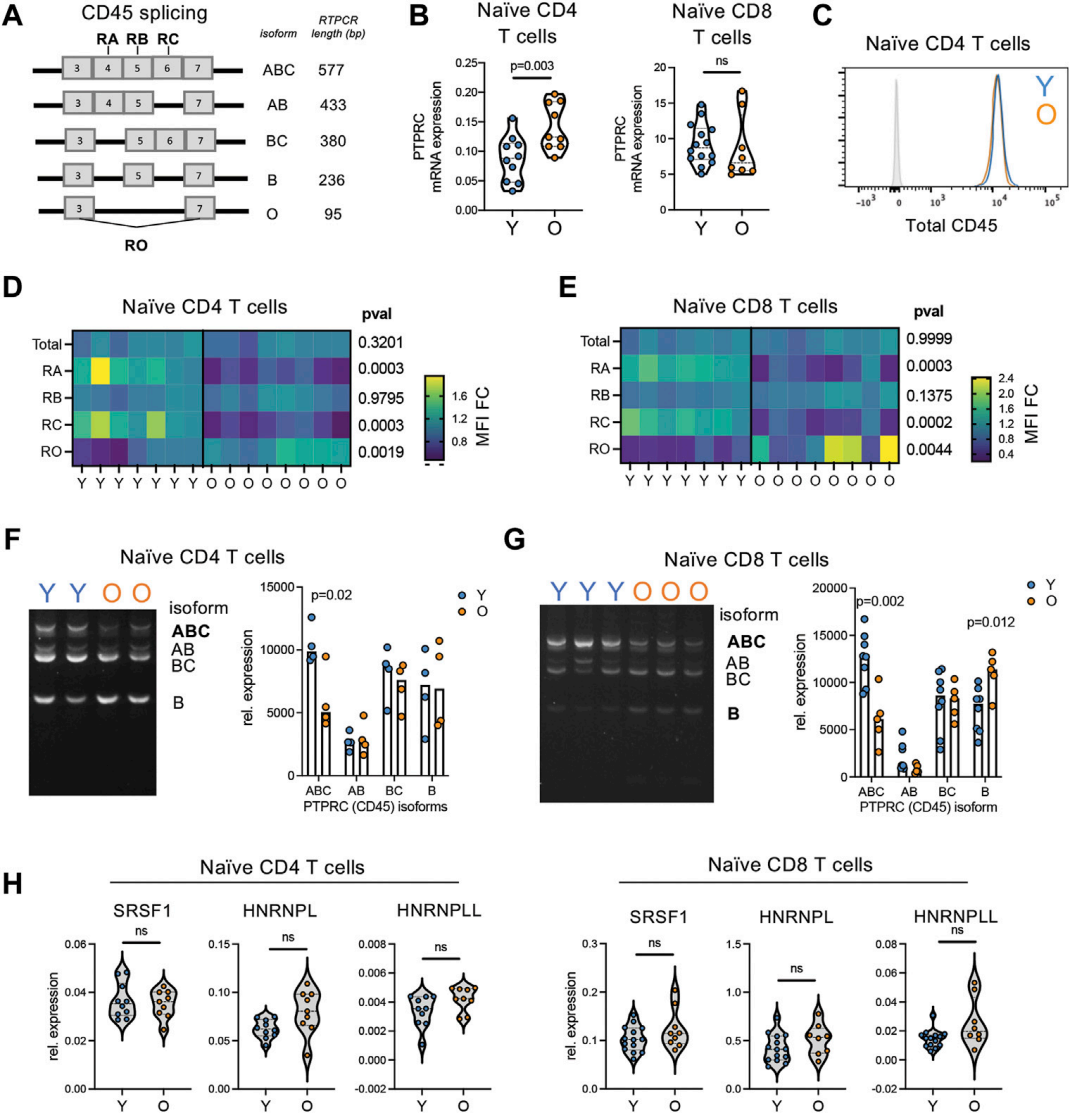
Reductions in CD45RA is caused by alternative splicing of CD45 not total protein loss. **(A)** Overview of CD45 isoforms. Bold indicated which exons specific antibodies bind to. **(B)** RNA expression of CD45 (PTPRC) in bulk naïve (CD45RA + CCR7+) CD4 and CD8 T cells from young (*n* = 10) and older (*n* = 9) adults. **(C)** Representative flow histogram of CD45 protein expression on naïve CD4 T cells from a young (blue) and older (orange) adult. Gray is negative control. **(D)** Naïve CD4 and **(E)** naïve CD8 T cell expression of total protein levels for CD45, CD45RA, −RB, −RC, and −RO in young (*n* = 7) and older (*n* = 8) adults detected by antibody. **(F)** Naïve CD4 and **(G)** naïve CD8 T cell expression of CD45 isoforms *via* RT-PCR analysis. Cell input was normalized for total CD45 expression. CD45RO isoform expression was too low in naïve T cells to be accurately quantified. **(H)** Expression of potential CD45 splice factors in naïve CD4 and CD8 T cells from young (*n* = 10) and older (*n* = 9) adults. *p*-values determined by **(B,D,E,H)** Mann-Whitney test and **(F,G)** unpaired *t*-test.

The loss of CD45RA density detected by protein level could be caused by two different molecular mechanisms; by the decrease in total CD45 or by alternative usage of CD45 isoforms (Akbar et al., 1988; Merkenschlager and Beverley 1989). Thus, we next interrogated CD45 splicing in naïve T cell from young and older adults. An overview of CD45 isoform expression and expression profiles on T cell subsets is provided in Figure 8A and Supplementary Figure S4. Both transcriptionally and by protein levels, no decrease in expression of total CD45 (encoded by *PTPRC*) was found with age in the naïve compartments (Figures 8B,C). Indeed, CD45 was higher in naïve T cells from some older individuals compared with young adults. However, in our analysis of CD45 *via* the expression of different exons using exon-specific antibodies, we found that both CD45RA (exon 4) and CD45RC (exon 6) protein densities decreased with age, although remaining above the negative threshold. CD45RB (exon 5) remained stable whereas CD45RO (lack of exons 4, 5, and 6) slightly increased (Figures 8D,E). CD45RO antibody is classically used to determine naïve from memory T cells *via* CD45RO^high/+^CD45RA^neg^ expression (Supplementary Figure S4). Of note, the CD45RO expression by naïve T cells from older individuals was significantly lower than that of memory T cells. Together these data indicate a change in isoform usage rather than a loss of CD45.

We next quantified the expression of specific CD45 isoforms at an RNA level using RT-PCR. A representative example of PTPRC (CD45 gene) isoform expression profiles across T subsets is provided in Supplementary Figure S5. Using this RT-PCR method, we found higher expression of the high molecular weight isoform CD45ABC in naive T cells from young adults (Figures 8F,G). This is consistent with the increased expression of CD45RA and CD45RC found in young individuals *via* flow cytometry (Figures 8D,E; Supplementary Figure S4). Moreover, this is consistent with flow cytometry analysis of naïve CD4 and CD8 T cells within organoids, which exhibit increased levels of CD45RA and CD45RC compared to 2DF conditions without increased CD45RO (Supplementary Figure S4C). To further investigate alternative splicing, we determined expression levels of three known CD45 alternative splice factors, HNRNPL, HNRNPLL and SRSF1. No consistent increase in these splice factors was found with age across naïve CD4 and CD8 T cells (Figure 8H). Thus, conversion to CD45RA^low^ phenotype with age is caused by alterative splicing of CD45 *via* the loss of the high-molecular weight CD45RABC isoform but not *via* increased expression of classical activation/differentiation-related splicing factors.

## 4 Discussion

“Naïve” T cells are often considered a single population however, previous aging research has demonstrated the acquisition of naïve-like memory cells within the naïve compartment with age, and many argue that these memory populations are driving the age-related difference observed in naïve T cells. Here, we reveal that a classically CD45RA^+^CCR7^+^ defined naïve compartment phenotypically changes with age, converting from a CD45RA^high^ CD27^high^CD25^neg^ to a CD45RA^low^CD45RO^low/neg^ population with age. Importantly, this conversion occurs within the antigen-inexperienced population, highlighting that this age-related change is independent from infiltration of the naïve compartment with naïve-like or stem cell memory cells. This finding is also consistent with our previous finding that there are global changes in miRNA expression in naïve CD8 T cells in older individuals (Gustafson et al., 2019). Thus, naïve T cells not only numerically decline but also phenotypically shift with human aging.

The partial downregulation of CD45RA on naïve T cells, along with the overall expansion of central memory T cells with age is indicative of partial conversion of these cells. This finding is reminiscence of previously described virtual memory cells (T_VM_), which are naïve CD8 T cells that acquire memory phenotype due to cytokine-induced proliferation in the absence of antigen-specific stimulation (White et al., 2016). T_VM_ cells make up around 10%–20% of the naïve population in mice (Akue, Lee, and Jameson 2012; Sosinowski et al., 2013) but expand with age (Chiu et al., 2013). However, in mice virtual memory cells are classically of a central memory phenotype and not within the naïve compartment. Thus, it is possible that the phenotypic shifts we observe with aging in humans is reflective of the development of cells into a virtual memory phenotype and potentially indicates that CD45RA and CD27 densities may provide effective markers for the identification of human T_VM_ cells.

The most prominent marker of “youthful” naïve cells was the high expression of CD45RABC isoform of CD45. The loss of this specific isoform in the absence of total CD45 changes has potential functional implications. CD45 modulates T cell activation thresholds (Courtney et al., 2019) and its total level of the surface of T cells is linked with cell proliferation and cytokine production (Dawes et al., 2006). Less is known about the function of different isoforms of CD45; however, humans with point-mutations in CD45 that change its splicing display altered susceptibility to autoimmunity and viral infections (Boxall et al., 2004). Humans with the C77G mutation cannot excise exon 4 (detected by CD45RA antibody), thus all cells retain expression of higher molecular weight isoforms. T cells in these individuals are more responsive to IL-2-induced proliferation (Windhagen et al., 2007), however Tregs within these patients have lower TCR-mediated activation and reduced suppressive function. Similarly, A138G mutation, which promotes CD45 splicing towards low molecular weight isoforms, reduces the ability of CD8 T cells to produce IFN-gamma (Boxall et al., 2004). These data suggest that alternations in expression patterns of CD45 isoforms may influence the function of naïve T cells and their ability to respond to antigen. Altered splicing of CD45 in naïve T cells with age also begs the question of whether there are more global splicing changes in these cells. A new study identified that LINE1-regulated splicing modulates naïve T cell quiescence (Marasca et al., 2022). As we observed no expression changes in classical CD45 splice factors and LINE1 is found within an intronic region of PTPRC, its role in regulation of naïve T cell aging and alterative splicing also warrants further investigation.

Studying human naïve T cell homeostasis and its breakdown is difficult. However, the observation that youthful naïve cells are enriched in lymph nodes and in turn our newly developed SLT-like organoid allowed the maintenance of this subset provides a new and exciting tool for the study of T cell aging. If we consider that naïve T cells spend a lot of their time within lymph nodes, it may not be surprising that they dislike standard, unstructured 2D culturing methods. Indeed, we find that even with stromal cell support, naïve T cells still acquire an aged phenotype in 2D culture. Moreover, youthful naïve T cells additionally require the presence of FRCs within this 3D structure for maintenance. The requirement for an organoid (3D + stromal cells) for any long-term maintenance of the youthful phenotype has potential implications; 1) the maintenance of a youthful phenotype is an active process, 2) cellular crosstalk is essential but not sufficient for this maintenance and 3) structural integrity of LN is vital for T cell homeostasis. Although the organoid formation seeds T cell and FRCs into the environment at the same, which is somewhat distinct from true LN homeostasis where the FRC network is already in place (Kelch et al., 2019; Horsnell et al., 2022), our data imply that FRC may change their behavior in an environment with structural breakdown, providing different, less homeostatic signals.

Mouse studies demonstrate that the maintenance of naïve T cell quiescence requires two different signals from local stromal cells; positive survival signal (i.e., IL-7) and negative inhibitory signal to prevent bystander activation (Siegert et al., 2011; Yu et al., 2017; Knoblich et al., 2018). Studies suggest that FRCs maintain their ability to secrete IL-7 with age (Thompson et al., 2019), however it is unclear whether alterations in the stromal cell inhibitory signals could contribute to phenotypic changes in naïve T cell homeostatic breakdown. However, we found no effect of FRC aging on naïve T cell phenotype or survival, implying that both the positive and negative signals coming from FRCs are unaffected by age. Similarly, a detailed analysis of lymph node FRCs revealed similar T cell-suppressive function of FRCs from young and older mice (Masters et al., 2019; Kwok et al., 2022). Alternatively, there is evidence of LN architecture breaks down. In mice and non-human primates, LNs have increased collagen deposition with age, which could affect structural integrity (Kwok et al., 2022). In human LNs, multiple changes have also been documented that could affect structural integrity and stromal cell behavior, such as fibrosis and lipomatosis (Cakala-Jakimowicz et al. 2021). Fibrosis can lead to physical reduction in T cell-FRC interactions required for homeostasis (Kwok et al., 2022), which may be mimicked in our 2DF culturing with reduced likelihood of T cell-FRC interactions compared to a 3D microenvironment of the organoids. However, our understanding of the specific differences in stromal cell function and/or signals from direct contact within an altered tissue architecture remains incomplete. Moreover, other cell types present within LN may contribute to T cell homeostasis, such as marginal reticular cell and endothelial cell interactions (Saxena et al., 2019). Our organoid model has the capacity to maintain multiple different cell types within the structure. Therefore, further investigation into the ability of different cell compartments to maintain T cell homeostasis in our organoids as well as the interactions between LN microenvironment, structural cells and immune cells during homeostasis and with age are warranted to provide better insight into the extrinsic causes of naïve T cell aging.

## Data Availability

The raw data supporting the conclusion of this article will be made available by the authors, without undue reservation.
