# Supplementary material for: The influence of three-dimensional structure on naïve T cell homeostasis and aging
Source: Front Aging. 2022 Nov 7;3:1045648. doi: 10.3389/fragi.2022.1045648 (PMC9676450; doi:10.3389/fragi.2022.1045648)
Supplement: Supplementary file 1 [file DataSheet1.docx]

**Supplemental Materials:** The influence of three-dimensional structure on naïve T cell homeostasis and aging

**
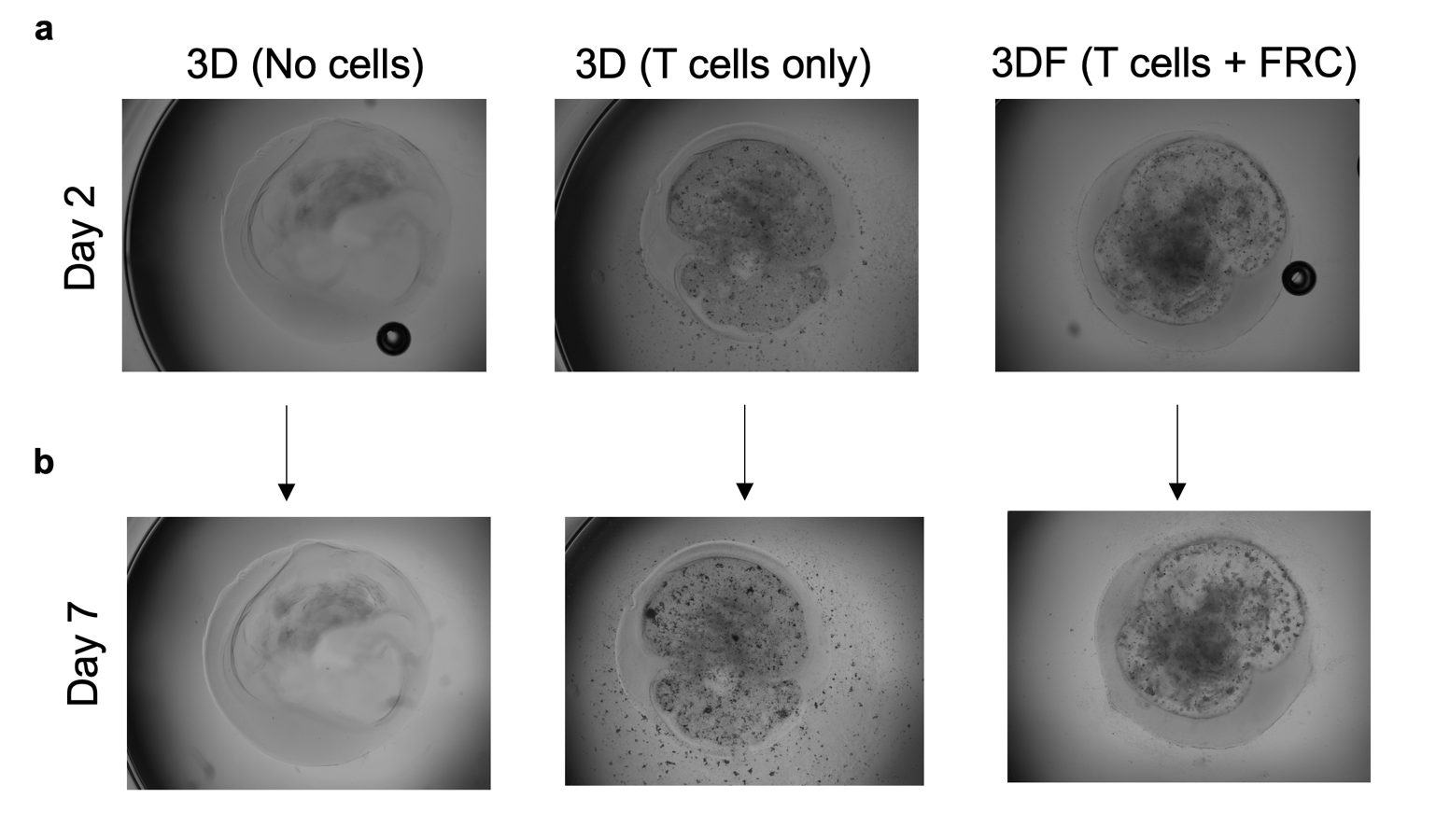
**

**Supp Figure 1. Whole organoid images over time. a-b)** Representative 4x magnification (phase contrast) images of organoids **at a)** day 2 and **b)** day 7 of culturing, taken on a EVOS M5000.

**
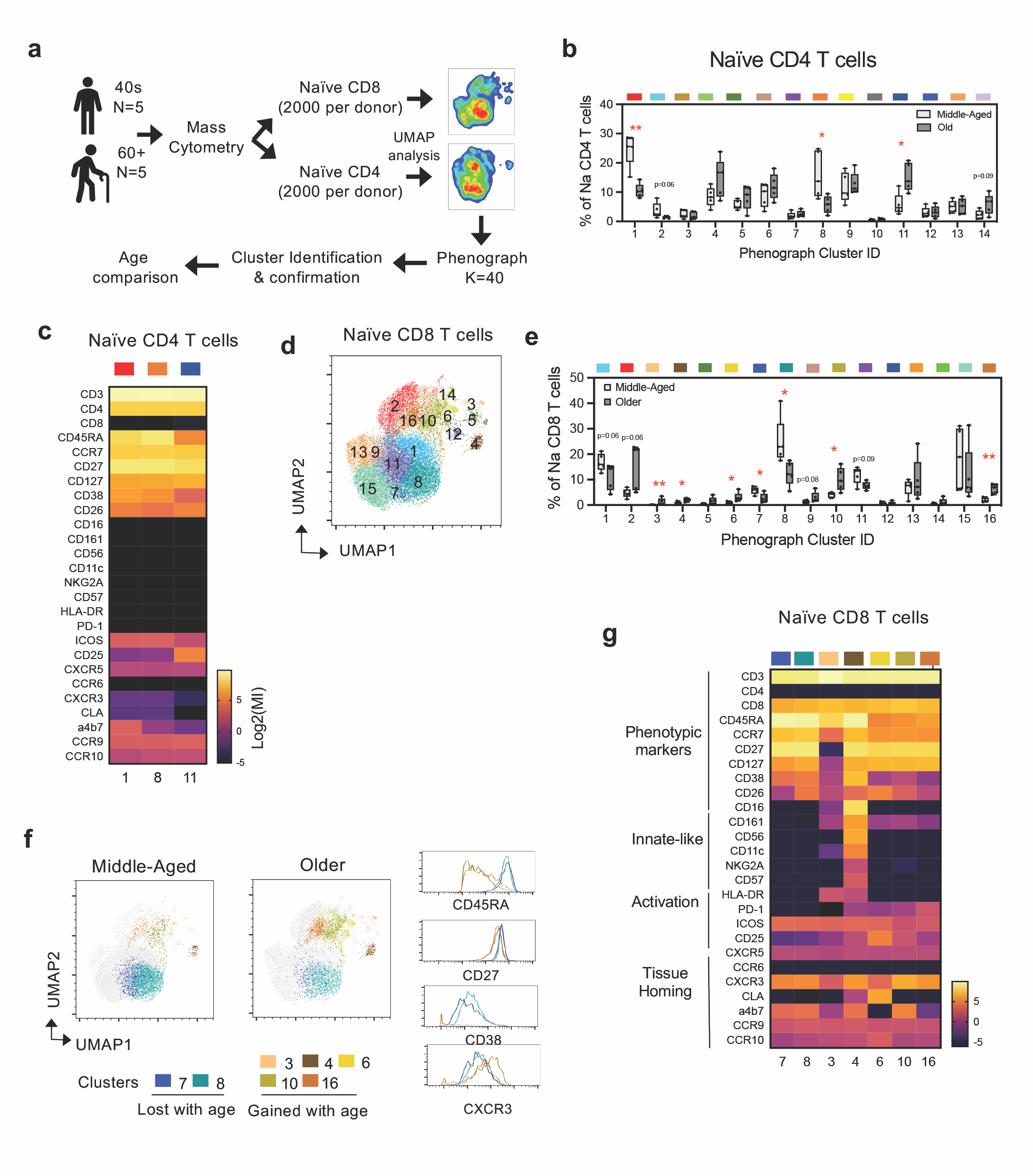
**

**Supp Figure 2. Mass cytometry analysis of the surface proteome of naïve T cell across age. a)** Mass cytometry experimental overview**. b)** Naïve CD4 T cell cluster frequencies in middle-aged (n=5) and older (n=5) individuals. q-values shown. **c)** Heatmaps of marker expression on differential clusters between middle-age and older adult naïve CD4 T cells. **d)** UMAP of Phenograph clusters in middle-aged and older adult naïve CD8 T cells. **e)** Naïve CD8 T cell cluster frequencies in middle-aged (n=5) and older (n=5) individuals. q-values shown. **f)** UMAP of differential clusters between middle-age and older adult naïve CD8 T cells with histograms of select markers delineating age-related clusters. **g)** Heatmaps of marker expression on differential clusters between middle-age and older adult naïve CD8 T cells.

**
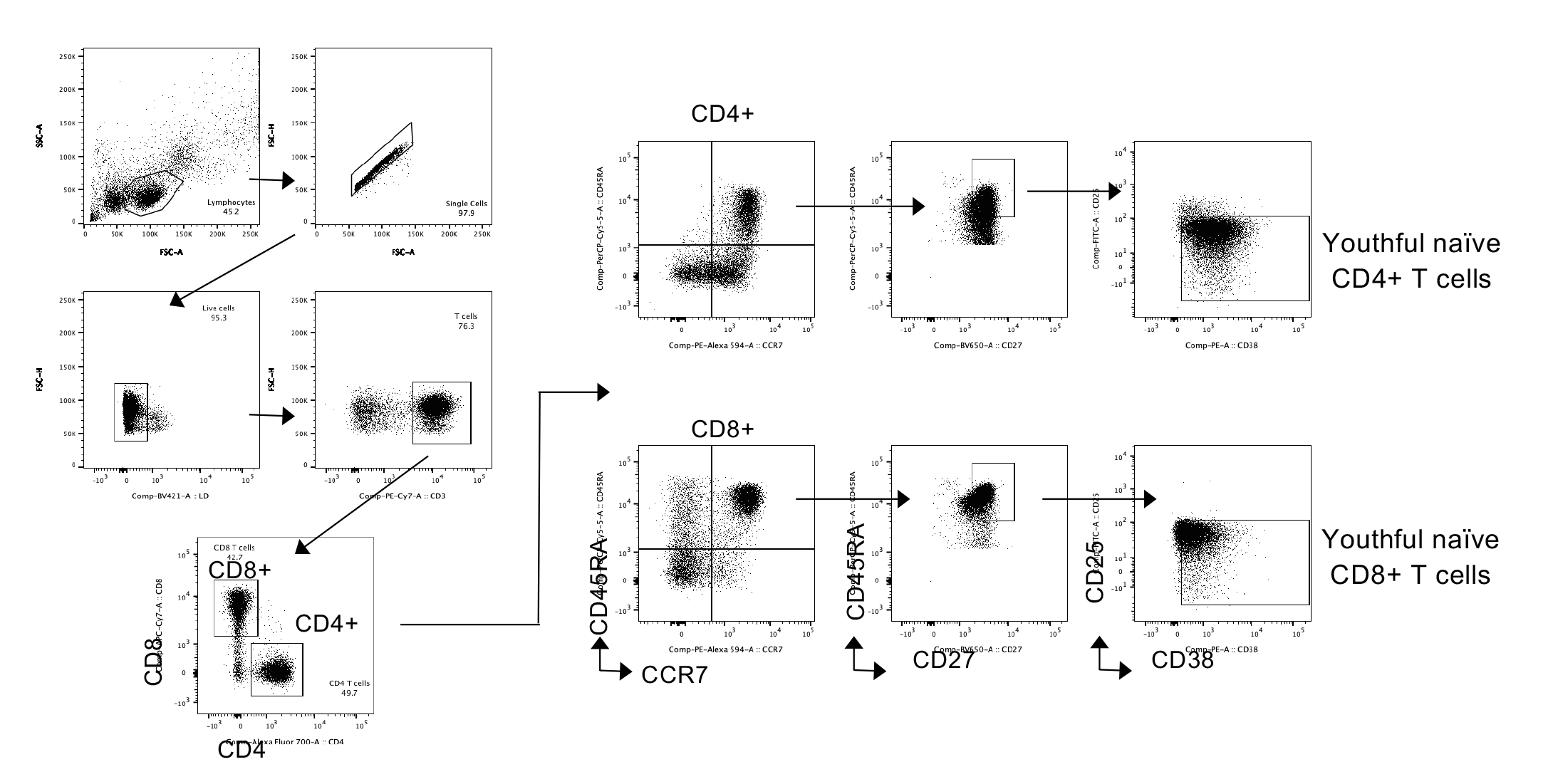
**

**Supp Figure 3. Gating of youthful naïve T cells using flow cytometry.**

**
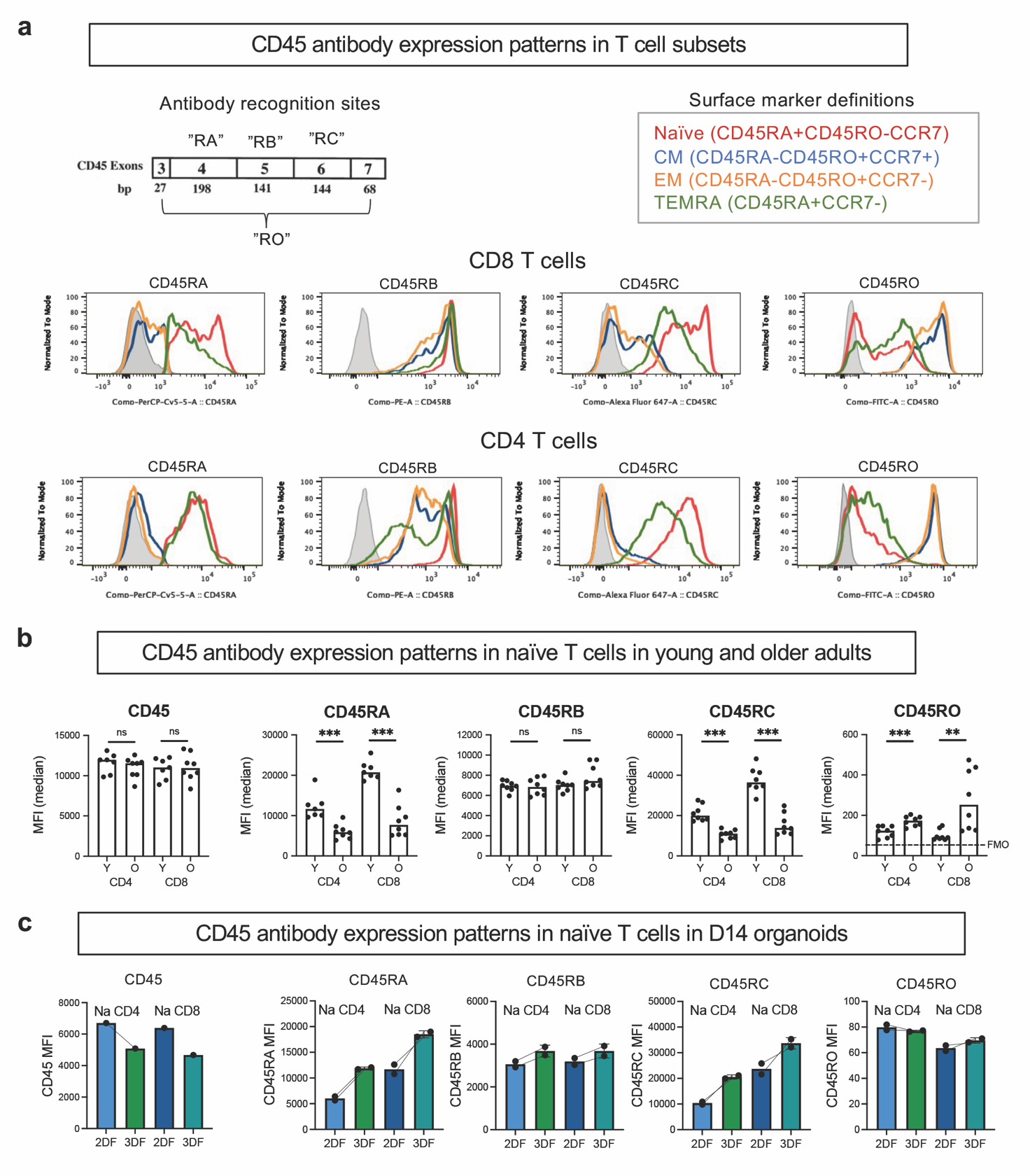
Supp Figure 4. CD45 antibody expression patterns across T cell subsets and by age. a)** Flow cytometry was used to examine expression profiles of CD45RA (exon 4), CD45RB (exon 5), CD45RC (exon 6) and CD45RO (exon junction of exon 3 and 7) at a protein level across naïve, central memory (CM), effector memory (EM) and TEMRA populations. **b)** Median fluorescence intensity comparisons of expression levels in naïve CD4 and CD8 T cells from young (n=7) and older (n=7) adults. P-values determined by Mann-Whitney test. **c)** The expression of CD45 and CD45R antibodies on naïve T cells (n = 1 young T cell donor, 1-2 replicates) after 14 days culturing in 3DF organoids or 2DF conditions.

**
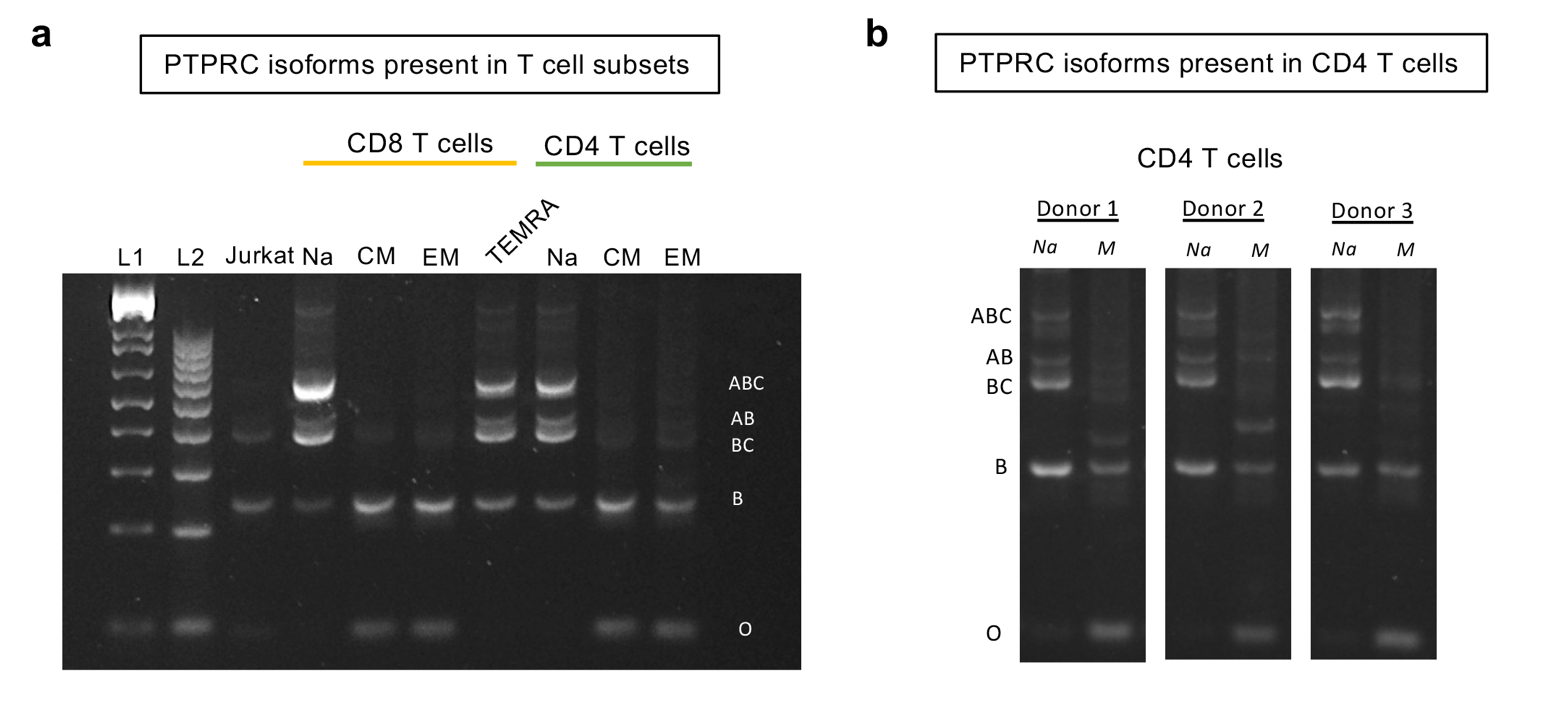
**

**Supp Figure 5. CD45 isoform comparison across T cell naïve and memory subsets. a)** RT-PCR was used to detect specific PTPRC (CD45 gene name) isoforms via RNA expression across T cell subsets and Jurkat T cell line. **b)** PTPRC isoform expression profiles in naïve (CD45RA^+^) and memory (CD45RA^neg^) CD4 T cells across three different donors.

**Supplemental Table 1. Donor Information for Tonsil tissue and Mass cytometry experiments.**

| **A) FRC Aging (Figure 6)** | | |
| --- | --- | --- |
| Tonsil Donor | Age | Sex |
| YA1 | 27 | Female |
| YA2 | 31 | Male |
| YA3 | 41 | Male |
| OA1 | 85 | Female |
| OA2 | 94 | Male |
| OA3 | 96 | Male |
|  |  |  |
|  |  |  |
| **B) Mass Cytometry (Figure 4)** | | |
| Blood Donor | Age | Sex |
| MA1 | 45 | Female |
| MA2 | 41 | Female |
| MA3 | 49 | Female |
| MA4 | 46 | Female |
| MA5 | 47 | Male |
| OA1 | 70 | Female |
| OA2 | 67 | Female |
| OA3 | 70 | Male |
| OA4 | 72 | Female |
| OA5 | 76 | Male |

**Supplemental Table 2. Antibodies used in these studies.**

| **Reagent type or resource** | **Designation** | **Clone (If applicable)** | **Source or reference** | **Catalog number** | **RRID, if available** | **Comments** |
| --- | --- | --- | --- | --- | --- | --- |
| **Mass cytometry panel** | | | | | | |
| Antibody | CD57-113In | HCD57 | HIMC | n/a | RRID:AB_2563757 |  |
| Antibody | HLA-DR-115In | G46.6 | HIMC | n/a | RRID:AB_396144 |  |
| Antibody | CCR6-Pr141 | G034E3 | Fluidigm | 3141003A | RRID:AB_2687639 |  |
| Antibody | CD19-Nd142 | HIB19 | Fluidigm | 3142001B | n/a |  |
| Antibody | IgG-Nd144 | G18-145 | HIMC | n/a | RRID:AB_398478 |  |
| Antibody | CD4-Nd145 | RPA-T4 | Fluidigm | 3145001B | n/a |  |
| Antibody | IgD-Nd146 | IA6-2 | Fluidigm | 3146005B | RRID:AB_2811082 |  |
| Antibody | CD20-Sm147 | 2H7 | Fluidigm | 3147001B | RRID:AB_2921324 |  |
| Antibody | IgA-Nd148 | G18-1 | HIMC | n/a | RRID:AB_396198 |  |
| Antibody | CD56-Sm149 | NCAM16.2 | Fluidigm | 3149021B | n/a |  |
| Antibody | CD86-Nd150 | IT2.2 | Fluidigm | 3150020B | n/a |  |
| Antibody | ICOS-Eu151 | C398.4A | Fluidigm | 3151020B | n/a |  |
| Antibody | TCRgd-Sm152 | 11F2 | Fluidigm | 3152008B | RRID:AB_2687643 |  |
| Antibody | CD45RA-Eu153 | HI100 | Fluidigm | 3153001B | RRID:AB_2802108 |  |
| Antibody | CD123-Sm154 | 9F5 | HIMC | n/a | RRID:AB_395999 |  |
| Antibody | CD27-Gd155 | L128 | Fluidigm | 3155001B | RRID:AB_2687645 |  |
| Antibody | CXCR3-Gd156 | G025H7 | Fluidigm | 3156004B | RRID:AB_2687646 |  |
| Antibody | NKG2A-Gd158 | S19004C | HIMC | n/a | RRID:AB_2888861, RRID:AB_2563787 |  |
| Antibody | CD11c-Tb159 | Bu15 | Fluidigm | 3159001B | n/a |  |
| Antibody | CD14-Gd160 | M5E2 | Fluidigm | 3160001B | RRID:AB_2687634 |  |
| Antibody | CD26-Dy161 | BA5b | Fluidigm | 3161015B | n/a |  |
| Antibody | CD8a-Dy162 | RPA-T8 | Fluidigm | 3162015B | RRID:AB_2811089 |  |
| Antibody | CD33-Dy163 | WM53 | Fluidigm | 3163023B | n/a |  |
| Antibody | CD161-Dy164 | HP-3G10 | Fluidigm | 3164009B | RRID:AB_2687651 |  |
| Antibody | CD127-Ho165 | A019D5 | Fluidigm | 3165008B | RRID:AB_2868401 |  |
| Antibody | CCR10-Er166 | 1B5-APC | HIMC | n/a | RRID:AB_2738943, RRID:AB_2563706 |  |
| Antibody | CCR7-Er167 | G043H7 | Fluidigm | 3167009A | RRID:AB_2858236 |  |
| Antibody | CCR9-Er168 | L053E8 | Fluidigm | 3168011A | n/a |  |
| Antibody | CD25-Tm169 | 2A3 | Fluidigm | 3169003B | n/a |  |
| Antibody | CD3-Er170 | UCHT1 | Fluidigm | 3170001B | RRID:AB_2811085 |  |
| Antibody | CXCR5-Yb171 | RF8B2 | Fluidigm | 3171014B | RRID:AB_2858239 |  |
| Antibody | CD38-Yb172 | HIT2 | Fluidigm | 3172007B | RRID:AB_2756288 |  |
| Antibody | a4b7-Yb173 | Act1 | HIMC | n/a | n/a |  |
| Antibody | PD-1-Yb174 | EH12.2H7 | Fluidigm | 3174020B | RRID:AB_2868402 |  |
| Antibody | CD62L/L-selectin-Lu175 | DREG200 | HIMC | n/a | n/a |  |
| Antibody | CLA-Yb176 | HECA-452 | Fluidigm | 3176018B | n/a |  |
| Antibody | CD16-209Bi | 3G8 | Fluidigm | 3209002B | RRID:AB_2756431 |  |
| **Flow Cytometry Antibodies** | | | | | | |
| Antibody | podoplanin-PE-Cy7 | NC-08 | Biolegend | 337014 | RRID:AB_2563368 | FRC panel |
| Antibody | CD31-AF647 | M89D3 | BD | 558094 | RRID:AB_397020 | FRC panel |
| Antibody | CD45-PE | 2D1 | Biolegend | 368509 | RRID:AB_2566369 | FRC panel, splicing panel |
| Antibody | CD4-AF700 | SK3 | Biolegend | 344622 | RRID:AB_2563150 | organoid panel, splicing panel |
| Antibody | CD45RA-APC | HI100 | Biolegend | 304112 | RRID:AB_314416 | organoid panel |
| Antibody | CCR7-PE | G043H7 | Biolegend | 353204 | RRID:AB_10913813 | organoid panel |
| Antibody | CD8-APC-Cy7 | RPA-T8 | Biolegend | 301016 | RRID:AB_314134 | organoid panel, splicing panel |
| Antibody | CD3-PE-Cy7 | UCHT1 | Biolegend | 300420 | RRID:AB_439781 | organoid panel, splicing panel |
| Antibody | CCR7-PE | G043H7 | Biolegend | 353204 | RRID:AB_10913813 | organoid panel |
| Antibody | CD27-PerCpCy5.5 | O323 | Biolegend | 302820 | RRID:AB_2073318 | organoid panel |
| Antibody | CD45RB-PE | MEM-55 | Biolegend | 310204 | RRID:AB_314807 | splicing panel |
| Antibody | CD45RC-AF647 | MT2 | BD | 565857 | RRID:AB_2869721 | splicing panel |
| Antibody | CD45RO-FITC | UCHL1 | Biolegend | 304204 | RRID:AB_314420 | splicing panel |
| Antibody | CD27-BV650 | O323 | Biolegend | 302828 | RRID:AB_2562096 | splicing panel |
| Antibody | CD45RA-PerCpCy5.5 | HI100 | Biolegend | 304122 | RRID:AB_893357 | splicing panel |
| Antibody | CD3-PE | HIT3a | BD | 555340 | RRID:AB_395746 | tetramer panel |
| Antibody | CD8-FITC | RPA-T8 | BD | 555366 | RRID:AB_395769 | tetramer panel |
| Antibody | CD45RA-BV605 | HI100 | Biolegend | 304134 | RRID:AB_2563814 | tetramer panel |
| Antibody | CCR7-PerCpCy5.5 | G043H7 | Biolegend | 353220 | RRID:AB_10916121 | tetramer panel |

**Supplemental Table 3. Taqman assays used in these studies.**

| **Gene** | **Assay ID** | **Figure** |
| --- | --- | --- |
| PTPRC | Hs04189704_m1 | 8 |
| HNRNPL | Hs00704851_g1 | 8 |
| HNRNPLL | Hs00293181_m1 | 8 |
| SRSF1 | Hs00199471_m1 | 8 |
| CDKN2A (p16) | Hs00923894_m1 | 6 |
| CDKN1A (p21) | Hs00355782_m1 | 6 |
| IL6 | Hs00174131_m1 | 6 |
| RPLP0 | Hs99999902_m1 | 6, 8 |
